# FedDroidMeter: A Privacy Risk Evaluator for FL-Based Android Malware Classification Systems

**DOI:** 10.3390/e25071053

**Published:** 2023-07-12

**Authors:** Changnan Jiang, Chunhe Xia, Zhuodong Liu, Tianbo Wang

**Affiliations:** 1 Key Laboratory of Beijing Network Technology, Beihang University, Beijing 100191, China; jcnby@buaa.edu.cn (C.J.); xch@buaa.edu.cn (C.X.); 16231285@buaa.edu.cn (Z.L.); 2 Guangxi Key Lab of Multi-Source Information Mining and Security, Guangxi Normal University, Guilin 541004, China; 3 Shanghai Key Laboratory of Computer Software Evaluating and Testing, Shanghai 201112, China; 4 School of Cyber Science and Technology, Beihang University, Beijing 100191, China

**Keywords:** federated learning, malware classification, privacy risk, sensitive information

## Abstract

In traditional centralized Android malware classifiers based on machine learning, the training sample uploaded by users contains sensitive personal information, such as app usage and device security status, which will undermine personal privacy if used directly by the server. Federated-learning-based Android malware classifiers have attracted much attention due to their privacy-preserving and multi-party joint modeling. However, research shows that indirect privacy inferences from curious central servers threaten this framework. We propose a privacy risk evaluation framework, FedDroidMeter, based on normalized mutual information in response to user privacy requirements to measure the privacy risk in FL-based malware classifiers. It captures the essential cause of the disclosure of sensitive information in classifiers, independent of the attack model and capability. We performed numerical assessments using the Androzoo dataset, the baseline FL-based classifiers, the privacy-inferred attack model, and the baseline methodology of privacy evaluation. The experimental results show that FedDroidMeter can measure the privacy risks of the classifiers more effectively. Meanwhile, by comparing different models, FL, and privacy parameter settings, we proved that FedDroidMeter could compare the privacy risk between different use cases equally. Finally, we preliminarily study the law of privacy risk in classifiers. The experimental results emphasize the importance of providing a systematic privacy risk evaluation framework for FL-based malware classifiers and provide experience and a theoretical basis for studying targeted defense methods.

## 1. Introduction

### 1.1. Background and Motivation

Due to the popularity of Android devices and their open source, Android users are subject to a wide range of malware attacks. Scientists have used machine learning (ML) to detect new malware and attack vectors. These traditional ML methods require centrally collected user data to train malware classifiers [1]. However, the app training sample set uploaded by users reveals much personal information, such as demographic data, equipment, and equipment security status, indicating personal privacy if directly used for analysis [2]. Privacy laws and scandals over the misuse of user data by some service providers have primarily curtailed this centralized approach [3]. To solve the privacy concerns of users, an FL-based malware classification framework with privacy protection has come into being [4]. In the FL framework, each participant uses a global training model and shares only model parameters without uploading their private data to a third-party server, avoiding direct privacy breaches. However, in recent years, there have been some privacy inference attacks against federated learning, such as attribute inference attacks (AInf) [5], model extraction attacks (MExt) [6], model inversion attacks (MInv) [7], and member inference attacks (MInf) [8], which can infer a user’s original data information from shared model parameters, leading to indirect privacy leakage. The vulnerability of FL training to privacy attacks in CV and NLP has been demonstrated. However, the existing FL-based malware classification framework does not consider the impact of privacy inference attacks or propose corresponding mitigation schemes. It does not accord with the existing privacy protection law, which hinders its development and application. At the same time, with the advent and popularity of the ChatGPT chatbot, users have raised privacy concerns about central servers with increasingly powerful model reasoning capabilities. The potential purpose of the center’s training server is unknowable and unmanageable. For projects involving innovative technologies such as machine learning, section 35 of the GDPR makes a data-protection impact assessment (DPIA) mandatory [9]. A critical step in DPIA is identifying potential data threats and assessing how those threats might affect individuals. Therefore, a tool is urgently needed to assess the privacy risk of user training samples under the FL-based malware classification framework.

Therefore, in this work, we comprehensively analyze the system and adversary model of the FL-based malware classification framework and propose a privacy risk evaluation framework, FedDroidMeter, based on normalized mutual information. In this framework, we design the input and output elements for privacy risk calculation according to the existing privacy threat attack surface, the characteristics of FL-based Android malware classifiers, and user privacy requirements. To verify the effectiveness and applicability of the proposed FedDroidMeter, we use the Androzoo dataset and baseline classification model to measure the privacy risk of the FL-based malware classification system.

The experimental results show that our proposed FedDroidMeter can effectively measure the privacy risks of the classification model. It also warns that existing FL-based Android malware classification systems do not provide adequate privacy protection. Adversaries can effectively infer the label and attribute information to the client’s training dataset (for example, the type of application used by the client and the type of malware infection). In addition, by comparing the input feature type, the setting of the federal hyperparameter, and the setting of the privacy protection parameter, we preliminarily study the law of privacy disclosure risk in the existing classifiers. These experiments and findings highlight the privacy risks faced by existing FL-based malware classification models and the importance of providing a systematic privacy risk evaluation framework. At the same time, it provides experience and a theoretical basis for developers to study the mitigation measures of privacy leakage.

The rest of this article is organized as follows. Section 1 provides an overview of the FL-based malware classification model and privacy inference attacks in machine learning and its evaluation methods. In Section 2, we introduce the system model and describe corresponding limitations. In Section 3, we introduce the proposed privacy risk evaluation framework based on normalized mutual information, FedDroidMeter. In Section 4, we describe our experimental setup and results. Finally, in Section 5, we present our conclusions and future work.

### 1.2. Related Work

In this section, we first review the latest work in the FL-based malware classification framework field. Second, we introduce existing privacy inference attacks on federated learning Lastly, the latest work of privacy breach evaluation schemes based on federated learning is reviewed, and the limitations and improvement direction of existing privacy breach evaluation schemes under the task of malware classification are analyzed.

#### 1.2.1. Malware Classification Method Based on Federated Learning

Existing methods that use ML/DL to classify malware rely on the vast amount of high-quality available data from different clients to train the accurate global model. These models are then distributed to individual clients, or these clients upload their test data to the server for real-time behavior checking and malware classification. However, training data contains private information about user behavior, which will seriously affect user security and privacy once it falls into the hands of hackers [10].

To solve user privacy concerns in deep learning, Google has proposed federated learning, a collaborative learning approach that ensures privacy by storing it locally on the client [11]. In the federated learning (FL) approach, each client executes local training using its local ML classifier model to generate local weights. The client uploads these local weights to the FL server. The FL server runs an average calculation on these local features and returns a usable global model. In FL, sharing local weights rather than raw data ensures user privacy [12].

Taheri [13] proposed a robust FL-based framework, namely FedIIoT, for detecting Android malware on the Internet of Things. FedIIoT forms a robust cooperative training model by adjusting two GAN-based adversarial algorithms. Narendra [14] proposed a lightweight model based on a convolutional neural network (CNN), which used a call graph, N-gram, and image transformation to extract relevant features. In addition, the author designed an auxiliary classifier generative adversarial network (AC-GAN) to generate invisible data for training. Shukla [15] introduced a performance-aware FL framework to reduce the communication overhead of device-level computing. Singh [16] used the FL framework to train a web security model from user browsing data and shared it with a centralized server. Valerian [17] proposed a privacy-preserving framework for malware detection on the Internet of Things, and their aggregation function based on mean pruning was tested as a countermeasure against adversarial attacks.

Galvez [4] presented LiM, a federated learning algorithm that works without user supervision, using safe semi-supervised learning techniques.

However, the existing FL-based malware classification schemes only consider the classification performance, communication performance, and robustness of poisoning attacks, and require more consideration and research on the privacy disclosure of malware classifiers. It makes the existing FL-based malware classification scheme unable to ensure user data privacy and make users transparently aware of their privacy risks under the threat of multiple privacy inference attacks. It does not accord with existing privacy protection laws, and hinders user willingness to participate in collaborative training. Therefore, it is urgent to propose a privacy risk evaluation mechanism for an FL-based Android malware classification scheme.

#### 1.2.2. Existing Inferential Attack Methods on Federated Learning

There are four types of privacy inference attacks: attribute inference attack (AInf), model extraction attack (MExt), model inversion attack (MInv), and member inference attack (MInf).

Member inference attacks are designed to determine whether candidate data belongs to the training dataset of the target model. Shokri et al. [8] proposed the first membership inference attack method for the black-box ML model. They trained multiple shadow models to simulate the target model and then use multiple attack models for reasoning. Salem et al. later relaxed several key assumptions [18], using multiple shadow models, knowledge of the structure of the target model, and having the same distribution of data sets as the auxiliary knowledge. Using new insights on how overfitting occurs in deep neural networks, Fredrikson [19] showed how the model’s particular use of features provides evidence of membership for white-box attackers and demonstrates that such attacks are superior to previous black-box approaches. Shafran [20] used a new predictability error that can be calculated for each sample and does not require a training set. The membership error is used to achieve a higher precision of membership inference on many baseline models by subtracting the predictability error from the reconstruction error.

The attribute inference attack was first proposed by Ateniese [21]. An internal attacker trains a meta-classifier by collecting the intermediate results of the updated model to infer sensitive statistical attributes contained in the training dataset of other actors. Later, Melis [22] proposed the first sample-level attribute inference attack against federated learning systems. Song [23] revealed that the inference of risk attributes is caused by the overlearning characteristic inherent in ML models.

Model inversion is mainly divided into data reconstruction and training class inference. Model inversion was first proposed by Fredrikson [24] in the task of drug dose classification. Fredrikson proposed a new inversion attack method in the article [25], aiming to reconstruct representative samples for each type of target model. They extend model inversion to general ML settings using the backpropagation of the target ML model parameters. Carlini [26] showed that model inversion can also be performed efficiently for natural language processing models. In the article [27], the author proposed an attack method to infer participants’ private training data using the shared gradient. This method obtains approximately private training data by iteratively optimizing the “virtual” input and tags to minimize the distance between the virtual gradient and the natural gradient.

The ModSteal attack (ModSteal), also known as model extraction, targets the Extract parameters from the target model. Ideally, the opponent will be able to obtain a model with a very similar performance to the target model (the “stolen” model). Tramer et al. [28] proposed the first model-stealing attack against the black-box ML model. The training set is constructed by querying the target model, the similar model of the target model is trained, and the similar model is updated through continuous iterative training. Finally, the approximate model of the target model is extracted. Orekondy et al. [6] used a reinforcement learning-based approach to ask to what extent an adversary could steal the functionality of this “victim” model based only on black-box interactions, namely image input and predictive output. In addition, Seong et al. [29] proposed novel metamodel methods, Kennen, which can successfully predict attributes related to the architecture and training hyperparameters even under challenging scenarios.

These attacks have proven to be dangerous in the field of graphics and natural language processing. However, considering the characteristics of input and models in the field of malware classification, as well as the characteristics of Android user demand for privacy, they are different from them. In the FL-based malware classification framework, there has yet to be a thorough study of what user privacy information is contained and the extent to which it is exposed to various inferred attacks, nor has an effective defense scheme been proposed.

Therefore, it is urgent to evaluate the impact of various attacks and the degree of privacy leakage in the FL-base malware classification framework to provide support for its corresponding privacy protection scheme.

#### 1.2.3. Existing Privacy Evaluation Methods for Federated Learning

In Reference [30], the authors proposed a method to measure the leakage of training data for machine-learning models, which relies on the results of performing member inference attacks for risk evaluation. In Reference [31], the author used the mean square error (MSE) between the reconstructed and original images to quantify the amount of privacy information leakage. However, this evaluation method depends on the configuration of the reconstructed attack model and is only applicable to scenes sensitive to the reconstructed features such as image or voice. Based on privacy risk evaluation methods of inference attack results, researchers have developed privacy evaluation libraries, such as MLPrivacyMeter [32], proposed by Murakonda, and MLDoctor [33], proposed by Liu.

However, these privacy evaluation schemes based on the attack effect cannot provide users with a reasonable privacy evaluation perspective, because attackers are unknowable to users. In the article [34], the author proposed a measure of member privacy based on the Shapley value, which is the contribution of training data records to the model’s utility. Song et al. [35] introduced a new method for fine-grained privacy analysis through a new privacy risk indicator, which estimated the probability of a single sample in the target model training set. However, as a posterior measure, it cannot be a privacy risk evaluation method for data owners. Similarly, in [36], the author used Fisher Information, another measure of the utility of the training data to the model, to estimate members’ privacy risks. However, Fisher information is expensive to compute because it requires reversing the Hessian function. In addition, its computational paradigm is only applicable to capture the relevant information between the data members and the model but not applicable to capture the sensitive attribute information in the data. In Reference [37], the author proposed the maximum information leakage as a measure of member privacy risk, which is the privacy risk upper limit of the PAT differential privacy framework. However, this information leakage metric measures the amount of leakage of all information in the data and does not distinguish between sensitive and ordinary information. Similarly, Rassouli [38] proposed the total variation distance as a measure of privacy in information disclosure scenarios. The analysis of the total variational distance relies on the joint distribution of known private and available data. However, an accurate data distribution cannot be available in practice. Yu et al. [39] proposed a case-by-case privacy accounting method for DP-SGD-trained models. However, this method is unsuitable for comparing the privacy risks between different models and does not consider the privacy risks of specific sensitive user information. Yang [40] evaluated the privacy leakage risk from the data owners’ perspective by constructing a K-means clustering distance method based on training data but did not consider the influence of training model hyperparameters.

Most of these previous studies have explored the empirical privacy risks of the ML model from the attacker’s perspective, as shown in Table 1. However, the actual privacy risks generated by attackers setting different capabilities and attack models are different. In addition, the attacker’s capabilities and models are unknowable to users, which makes the inferred attack-based measures not applicable to users. on the other hand, some attempts at risk evaluation methods from the user perspective do not consider the influence of the classification model’s hyperparameters and privacy protection parameters; simultaneously, the sensitive information users care about in the dataset is not considered.

To sum up, existing privacy risk evaluation frameworks do not capture the essence of privacy leakage in the system, thus, cannot provide effective evaluation results for users of the federal Android malware classifiers.

Therefore, developing a user-oriented privacy risk evaluation framework for FL-based Android malware classifiers is necessary. The evaluation framework allows users to evaluate the potential privacy risks of their training samples throughout the life cycle of the FL-based Android malware classification model. The privacy risk evaluation framework needs to: (1) meet user-oriented privacy requirements; (2) be attack-agnostic; and (3) have an equal comparison between different use cases.

### 1.3. Contribution

The contributions of this paper are summarized below.

1. We comprehensively analyzed the privacy threats in the FL-based Android malware classification system, and defined the sensitive information, observable data, and attacker types in the system; a privacy risk evaluation framework, FedDroidMeter, was proposed to evaluate the privacy disclosure risk of the FL-based malware classifier systematically.
2. We propose a calculation method for privacy risk score based on normalized mutual information between sensitive information and observable data. We use the sensitivity ratio between layers of the gradient to estimate the overall privacy risk, appropriately mitigating the score calculation.
3. We validate the effectiveness and applicability of the proposed evaluation framework on the Androzoo dataset, two baseline FL-based Android malware classification systems, and several state-of-the-art privacy inference attack methods.

## 2. System Model and Problem Description

### 2.1. System Model

An FL-based Android malware classification system allows Android clients to keep the malware dataset locally and collaboratively train classification models, which means that any third party cannot access the user’s raw app samples [3]. The framework of federated learning consists of a server and multiple clients. The server in this work refers to the remote FL server, and the client is an Android client.

**Figure 1:**
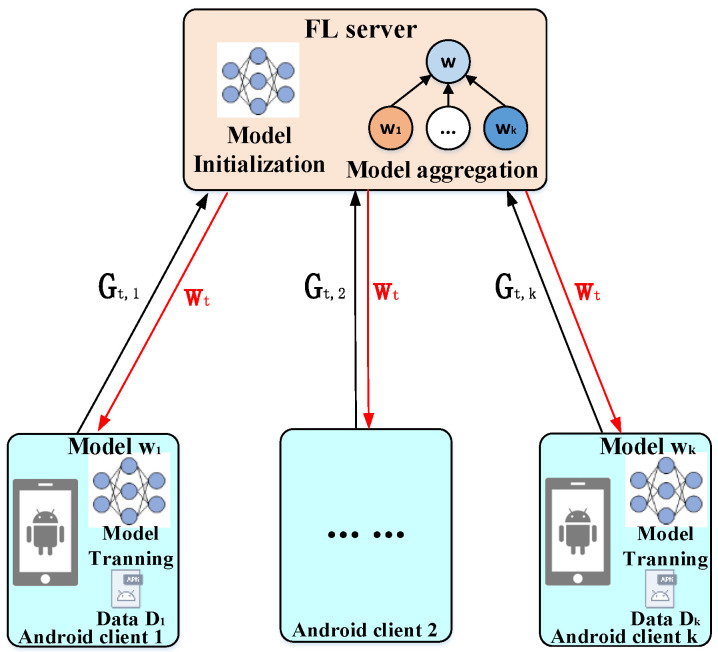
FL-based Android malware classification system.

We consider an FL-based Android malware classification system that includes one FL server for parameter aggregation and update server, and k Android clients have several training samples. The FL framework uses a given training algorithm (such as DNN) to collaboratively train a malware classification model. The overall structure of the FL-based system is shown in Figure 1.

There are the following stages in the process of federated learning: initialization stage, aggregation stage, and update stage, which are described as follows:

In the initialization phase, the FL server assigns a pre-trained global model $W_{t}$ to each Android client. Then, each client trains and improves the current global model $W_{t}$ using the local dataset $D_{k}$ of $D_{k}$ size in each iteration.

In the aggregation phase, the FL server collects local gradients of Android client uploads. The global loss function F(W) and local loss function $F_{k}(W)$ to be optimized in FL are shown in Formulas (1) and (2):

(1) $$\underset{w\in R^{d}}{\min}F(W)=\frac{\varphi_{k}}{\Upsilon }\sum_{k=1}^{N}F_{k}(W)$$

(2) $$\;F_{k}(W)=\frac{1}{\varphi_{k}}\sum_{z_{i}\in D_{k}}^{\;}\;f(W;z_{i})$$

where f(·) is the local loss function of Android client k,k∈[1,N], $z_{i}=\left(x_{i},y_{i},F_{i}\right),\;\forall i\in [1,\ldots ,\varphi_{k}]$ is sampled from the local dataset $D_{k}$ of k clients; Υ is the total number of global samples D, $\varphi_{k}$ is the number of samples of client k; $x_{i}\in X$ is the feature of Android malware, and $y_{i}\in Y$ is the category label of Android malware. In addition, $F_{i}\in F$ is the category of application functions of the app sample $z_{i}$.

(3) $${W^{t+1}}_{}\leftarrow {W_{}}^{t}+\frac{\varphi_{k}}{\Upsilon }\sum_{k=1}^{N}{\nabla_{{W^{t}}_{}}F}_{k}^{t}$$

In the update phase, the FL server uses the federated average algorithm (FedAvg) [12] to obtain a new global model $W_{t+1}$ for the next round, as shown in Formula (3). $\sum_{k=1}^{N}{\nabla_{{W^{t}}_{\;}}F}_{k\;}^{t}$ denotes the model update aggregation, ${{G_{t,k}=\nabla }_{{W^{t}}_{}}F}_{k}^{t}$ denotes the model’s gradient of k client, and $\frac{\varphi_{k}}{\Upsilon }\sum_{k=1}^{N}{\nabla_{{W^{t}}_{\;}}F}_{k\;}^{t}$ denotes average aggregation.

This process is repeated for both FL server and Android clients until the global model converges. By not requiring direct access to raw training data on Android nodes, this mode significantly reduces the risk of privacy disclosure.

### 2.2. Threat Model

**Figure 2:**
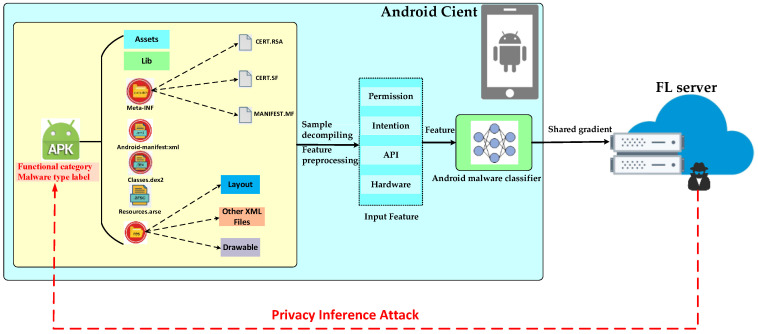
Threat Model of FL-based Android malware classification system.

This section presents several assumptions regarding adversaries who may attempt to infer privacy from the FL-based Android malware classification system, as shown in Figure 2.

(1) Role and capability of attack: Since we want to evaluate the worst case of privacy disclosure, we set the attacker as the FL server, which has sufficient auxiliary knowledge $D_{\mathrm{aux}}$ about the victim client (similarly distributed of app sample). The FL server models the main classification task through an FL scheme and aims to infer the sensitive information in a participant’s training sample from their shared parameters. The FL server honestly follows the FL process but is curious to infer sensitive information about the victim client. At the same time, we assume that all clients honestly follow the designed FL process. In addition, we assume that a public and private key pair has been shared, and the message cannot be forged or guessed ciphertext by an external attacker; That is, we only consider privacy threats from insider attackers.

(2) Target of the attacker: The attacker’s target is to infer the sensitive information of the app training sample of the victim client from the observable data in the FL-based malware classification framework through the privacy inference attack method.

(3) Attack methods: According to the summary in Section 2, the attacker can launch four types of training data privacy attacks: membership inference attacks, model inversion attacks, attribute inference attacks, and model-stealing attacks. At the same time, we assume that the attack mode is a white box because it has a more vital inference ability than the black-box attack [27]. The attacker knows all model parameters and hyperparameters in the FL procedure, including learning rate, local epoch, local batch size, and local sample characteristic type. It is important to emphasize that these four types of attacks represent the full spectrum of attack surfaces currently privacy-inferred for ML models of Android malware classification.

Next, we give formal definitions of the four most advanced inference attacks.

(1) Membership Inference attack (*MemInf*)

Membership inference attack aiming to detect whether or not a target sample was used to train a target ML model W. More formally, given a target sample ${(x}_{\mathrm{target}},y_{\mathrm{target}})$, (the access to) a target model W and gradient $G_{\;}$, and an auxiliary dataset $D_{\mathrm{aux}}$, a membership inference attack can be defined as Formula (4):

(4) $$\mathrm{MemInf}:\;[{(x}_{\mathrm{target}},y_{\mathrm{target}}),G/W,D_{\mathrm{aux}}]\to \{\mathrm{Member},\;\mathrm{Non}-\mathrm{Member}{\}}_{\;}$$

(2) Attribute inference attack (*AttrInf*)

Classification models based on machine learning will learn additional sensitive information in training data unrelated to the main task. The objective of an attribute inference attack is to detect (train by auxiliary dataset $D_{\mathrm{aux}}$) whether the unexpected sensitive information S carried in the model W and gradient $G_{\;}$. Formally, attribute inference is defined as Formula (5):

(5) $$AttrInf:\left[G/W,D_{\mathrm{aux}}\right]\to \{S,\bar{S}\}$$

(3) Model-stealing attack (*ModSteal*)

Model-stealing attacks aim to extract the parameters from a target model W. Ideally, an adversary will be able to obtain (train by auxiliary dataset $D_{\mathrm{aux}}$) a model $\hat{W}$ (the “stolen” model) with a very similar performance as the target model W, as shown in Formula (6):

(6) $$ModSteal:W,D_{aux}\to \hat{W}$$

where $\hat{W}$ is the stolen model.

(4) Model inversion attack (*ModInv*)

Model inversion attacks aim to reconstruct (train by auxiliary dataset $D_{\mathrm{aux}}$) training samples $\left\{\left(x_{i},y_{i}\right)\right\}$ from the target model’s gradient $G_{\;}$. Formally, we define model inversion as Formula (7):

(7) $$\mathrm{ModInv}:G_{\;},D_{\mathrm{aux}}\to \left\{\left(x_{i},y_{i}\right)\right\}$$

It should be emphasized that the actual privacy disclosure risk caused by these four privacy inference attacks on the users participating in the training needs to be further analyzed in combination with the privacy needs of users and the characteristics of the FL-based malware classifier, which will be analyzed in Section 3.2.

### 2.3. Problem Description

The above system model and threat analysis of the federated Android malware classifier shows that there are two privacy issues and challenges in the current system:

(1) An endless stream of privacy inference attacks against FL exists. However, the existing framework does not consider the indirect privacy disclosure caused by these attacks, leading to severe threats to users of app samples that provide training. Due to the variety of privacy inference attacks, and the complexity and opacity of the FL Android malware classification model, it is difficult for users to know what private information is leaked in the classification system and the source of privacy leakage.

(2) The FL-based Android malware classifier lacks a user-oriented privacy risk evaluation mechanism, so users cannot understand the degree and distribution of privacy disclosure risk in the classification system. It also makes privacy protection mechanism design for FL-based malware classifiers lack theoretical guidance and basis. Considering the characteristics of the FL-based Android malware classification system and the privacy needs of users, the existing privacy risk evaluation indicators and methods cannot provide effective evaluation results for the system.

These issues increase user privacy concerns and violate existing privacy laws (which violate GDPR principles of legality, fairness, and transparency by not letting users know about the legality of the training process), limiting the application and development of the federal Android malware classifier. It prompts us to deeply analyze the actual threat of privacy inference attacks to FL-based malware classification systems and user privacy needs, and develop a user-oriented privacy risk evaluation method for the FL-based Android malware classification system.

### 2.4. Goal of Design

The above issues increase user privacy concerns and violate existing privacy laws (which violate GDPR principles of legality, fairness, and transparency by not letting users know about the legality of the training process), limiting the application and development of the federal Android malware classifier. It prompts us to deeply analyze the actual threat of privacy inference attacks in FL-based malware classification systems and user privacy needs. Furthermore, we develop a user-oriented privacy risk evaluation method for the FL-based Android malware classification system.

The evaluation framework allows users to evaluate the potential privacy risks of their training samples throughout the life cycle of the FL-based Android malware classification system. The privacy risk evaluation framework needs to meet three principles: (1) User-oriented privacy requirements; (2) Attack-agnostic; and (3) Equal comparison between different use cases, as shown in Table 2. Among them, principles (1) and (2) ensure the effectiveness of privacy evaluation, and principle (3) ensures the applicability of privacy evaluation to different use cases.

## 3. Proposed Methods

In this section, we first introduce the components and functionality of FedDroidMeter, a proposed privacy risk evaluator for FL-based Android malware classifiers. Then, we introduced the design of FedDroidMeter’s input and output, respectively. The list of abbreviations and mathematical symbols used is given in Table 3.

### 3.1. FedDroidMeter Framework Overview

To solve the limitations of the existing framework proposed in Section 2.3, we designed a privacy risk evaluator, FedDroidMeter, to systematically evaluate the privacy disclosure risk of FL-based malware classifiers according to the privacy needs of users and the characteristics of scene threats.

**Figure 3:**
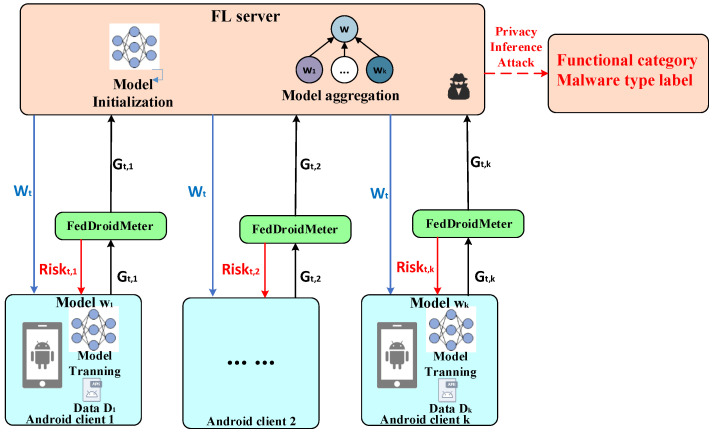
FedDroidMeter framework overview.

The system model for privacy risk evaluation in this article (as shown in Figure 3) mainly comprises three types of entities: an FL server, an Android client, and the proposed privacy risk evaluator FedDroidMeter.

(1) FL server: the FL server is a central server with strong computing power and rich computing resources. FL server contains two mechanisms, which: (a) initialize the global model and send the optimal global initialization model parameters to all Android clients; and (b) aggregate the gradient uploaded by Android clients until the model converges.

(2) Android client: the Android client has some samples of various types of malware, which is used for training on the model issued by the FL server, and then generates an updated gradient and sends it back to the server.

(3) FedDroidMeter: FedDroidMeter is a privacy risk evaluator deployed on the client to evaluate the privacy risks of the shared information that the client returns to the server. It takes observable data, adversary targets, and other parameters as input; the output of FedDroidMeter is the client’s privacy risk score.

The workflow of the FedDroidMeter comprises the following four steps:

(1) The Android client uses the initial model parameters $W^{t}$ of the Android malware classifier and the local dataset to calculate $G_{t,k}$, the updated gradient of model parameters;

(2) The FedDroidMeter (in Android clients) collects privacy-related elements in FL-based malware classifiers to evaluate the privacy risk score ${Risk}_{t,k}$ of the sharing gradient at this round. Then, the sharing gradient $G_{t,k}$ is uploaded to the FL server;

(3) The FL server aggregates the updated gradient $G_{t,k}$ of global model $W^{t}$ uploaded by k clients and updates the optimal global model parameters;

(4) The FL server sends new optimal global model parameters $W^{t+1}$ to each Android client. The above steps are repeated until the global model W reaches optimal convergence.

As described in Section 2, considering the diversity of privacy inference attacks and the actual scenario characteristics and privacy requirements in the FL-based malware classifier scenario, we need specific analysis on how to design privacy risk evaluators that meet the design goals. Meanwhile, inspired by Reference [41], we consider the four key questions to systematically guide the privacy risk evaluator’s design process to ensure their applicability and effectiveness:

In the deep-learning models performing FL-based Android malware classification, given the training set of app samples, the shared gradient (based on the training app samples), the classification model structure and parameters, and the adversary (non-specific) information,

**Question** **1:** *Where are the sources of privacy leaks?*

**Question** **2:** *What kind of private information will be leaked?*

**Question** **3:** *What determines the occurrence of privacy leaks?*

**Question** **4:** *What is the level of privacy leakage risk?*

### 3.2. Design of Input for Privacy Risk Evaluator

**Figure 4:**
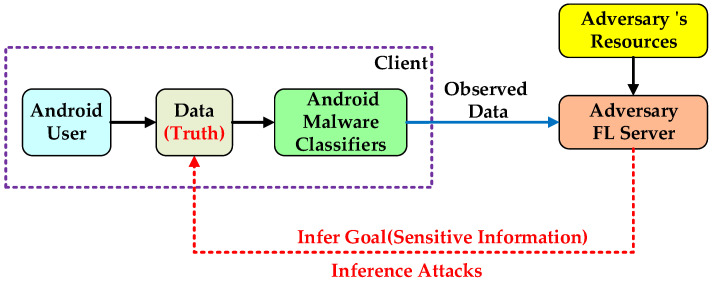
Mechanism of privacy disclosure in the threat model.

To evaluate privacy risks, the inputs to the evaluator should be relevant variables that reflect the underlying causes of privacy breaches in the system. Therefore, as shown in Figure 4, we conduct an in-depth analysis of the source and mechanism of privacy disclosure in the threat model (Answer **Question 1**).

In a given Android malware classification model, the adversary uses relevant auxiliary knowledge and privacy inference attack models to infer sensitive information about users in public data. Therefore, we designed the input elements of *FedDroidMeter* as shown in Formula (8):

(8) $$\begin{array}{c} {FedDroidMeter}_{Input}\\ =\left\{Infer\;Goal,Observed\;Data,Truth,adversary’s\;resources\right\} \end{array}$$

Among them, the adversary’s resources in the input setting are sufficient app sample sets with the same distribution as the inferred target client as auxiliary knowledge. In addition, the adversary has unconstrained computing power (from the perspective of privacy in information theory). In addition, the adversary can use a variety of advanced privacy inference attack models (selecting the most threatening results). These settings are used to evaluate the worst-case privacy risk while also obtaining attack-agnostic and effective evaluation results to ensure users’ privacy requirements. It should be pointed out that the attacker’s setting is also used to proofread the validity of the privacy score. In addition, Truth is the true answer about the opponent’s goal in the dataset. In the following sections, we will describe the definitions and settings of the remaining input elements.

#### 3.2.1. Analysis of Sensitive Information of User’s Training Data in FL-Based Android Malware Classifier

In this section, we provide a detailed definition and analysis of the sensitive information leaked to the attacker in the system, considering the various privacy threats brought by the attacker, the privacy requirements of Android users for training samples, and the characteristics of the FL-based Android malware classifier. (Answer to **Question 2**).

First, we define the sensitive information involving user privacy in the FL-based Android malware classification system.

**Definition** **1.** *Privacy is the ability of individuals or groups to hide their identity and information about themselves and then selectively express themselves.*

Privacy can be divided into two categories: identity privacy and attribute privacy [41]. In this article, we consider the attribute privacy of the user because, in an FL environment, the user’s ID is known (the Transparency and Accountability principles of the GDPR principles).

**Definition** **2.** *Attribute privacy is the ability of individuals or groups to hide information about themselves.*

Attribute privacy is a weaker privacy requirement than anonymous privacy assumption; a specific individual has been uniquely identified and needs to protect their personal information, such as user preferences and device security status.

**Definition** **3.** *Sensitive information is the information that may be inferred by adversaries in the FL-based Android malware classification system to violate the user’s attribute privacy.*

Based on the analysis of existing privacy inference attacks in the FL-based Android malware classification system, adversaries can infer the following elements:

(1) The functional class F of the training app samples.

(2) The malware class y of the training app samples.

(3) The input features x of the training app samples.

(4) The parameters of the trained global Android malware classification model W.

First, the function category F of the training app sample reflects the user’s usage information, and the category of the malware label reflects the security status information of the user’s device. The leakage of both violates the user’s attribute privacy.

Second, the input features of the training app samples are sensitive APIs, permissions, intentions, and bytecodes extracted from the app samples [1], which do not involve the user’s attribute privacy. In addition, these features cannot be returned to the source code (which would be a user’s copyright), so there is no violation of the user’s attribute privacy.

Finally, in the FL-based Android malware classification system, the global classification model is jointly trained by all clients, which can only further infer the general statistical information for all participants but cannot infer the information of specific clients. Therefore, the attribute privacy of users is not violated either.

In summary, in the FL-based Android malware classification system and its corresponding threat model, the sensitive information is the functional category F of the trained app samples and the category of the malware tags y of the trained app samples. Their corresponding attack modes are attributed inference attack and label inference attack, respectively.

#### 3.2.2. Analysis of Observable Data Sources of Training Data about Users in FL-Based Android Malware Classifiers

We find the observable data of attackers in each phase by analyzing the characteristics of the FL-based Android malware classification system in the whole cycle (data-processing phase, training phase, prediction phase), since the data-processing phase and prediction phase in the FL-based malware classification framework is carried out on the user device (we assume that the channel between the clients and the server is secure and there is no external attacker).

Therefore, the attacker has no observable data source that can be used for a privacy inference attack in both the data-processing and prediction phases. Thus, we focus on analyzing the observable data sources of the classification framework in the training phase.

In the training phase, the client samples the local user’s app sample set to obtain a batch of samples for training. The client then uploads the locally trained updated gradients. Among them, batch sampling rules for training can be roughly divided into three categories: random, systematic, and hierarchical.

Random sampling: The user randomly samples a certain amount of data from the dataset, which can be divided into two types: put back and not put back.

(1) Sampling method of putting back. Sample sampling probability as shown in Formula (9):

(9) $$P_{s}\left(i|D\right)=\frac{1}{\left|D\right|}$$

where $\left|D\right|$ is the size of the dataset. $P_{s}\left(i|D\right)$ is the probability of sampling the ith sample.

(2) Sampling method without putting back as shown in Formula (10).

(10) $$P_{s}\left(i|S_{e},D\right)=\frac{1}{\left|D\right|-\left|S_{e}\right|}\alpha \;\alpha =\left\{\begin{array}{c} 0,\;i\in S_{e}\\ 1,\;i\notin S_{e} \end{array}\right.$$

where $S_{e}$ is the sampled sample set, α is the indicator function. When the sampled sample is in $S_{e}$, the sampling probability is 0.

System sampling: The user divides the overall dataset into several small parts in order, then extracts the ith data from each small part.

Hierarchical sampling: Users divide data into several categories, then randomly select a certain number of samples from each category, and then combine these samples.

The training gradients generated by any two batches, whether randomly or systematically sampled, correspond to different subsets of app samples. Therefore, the attacker needs to run the analysis (train the inference model) in two separate batches to maximize inference accuracy. Thus, the observable data source for the attacker is a batch of generated update gradients. Accordingly, our metric framework computes the privacy risk score for the gradient of each batch separately.

Based on the above analysis, the input of the privacy risk evaluator *FedDroidMeter* as shown in Formula (11):

(11) $$\begin{array}{l} {FedDroidMeter}_{Input}\\ =\left\{Infer\;Goal,Observed\;Data,Truth,adversary’s\;resources\right\}\\ =\left\{{(F}_{\;},y),G_{t,k},\left(x,y,F\right),D_{\mathrm{aux}}\right\} \end{array}$$

### 3.3. Design of the Output of the Privacy Risk Evaluator

The evaluator’s output is the current system’s privacy risk level, calculated by various attributes (elements of the risk evaluator’s input) about the system and adversaries. Furthermore, to quantify the level of privacy risk, we need to design an appropriate privacy risk meter indicator and the corresponding calculation method of privacy risk score.

#### 3.3.1. Design of Output Privacy Risk Score

According to the three principles in the design objective of the privacy evaluation method, we choose the appropriate evaluation indicator. The principle of privacy requirements for users means that privacy indicators should reflect the disclosure of attribute privacy in the worst case. The principle of equal comparison between attack unknowability and instance means that the privacy evaluation indicator must capture the essential cause of privacy disclosure to reflect the degree of privacy risk in a way independent of the attack and use case. Therefore, we must dig deep into privacy disclosure’s essential causes to select effective evaluation indicators. From Section 3.2, we can see that the three factors related to privacy disclosure are the adversary’s strengths and objectives, observable data sources, and truth (the honest answer about the adversary’s objectives in the dataset).

Furthermore, the essential cause of privacy disclosure is a correlation between the object of the sensitive information (which the adversary is interested in) and the observable data source so that the adversary with a specific ability can use it for privacy inference (Answer the **Question 3**). It should be noted that the classification model of Android malware considered in this paper is a neural network (such as DNN and CNN). Therefore, the relationship between the observable data source (training gradient) and the adversary target (functional category and malware label category) is highly nonlinear, and it is tricky and impractical to directly derive the analytical mapping relationship from the gradient to the corresponding sensitive information.

The existing evaluation indexes do not measure this essential cause effectively but only approximately fit the correlation relationship from the side perspective of empirical attack results or the distribution distance or utility of training data, so it is challenging to obtain effective evaluation results. Therefore, we innovatively introduce normalized mutual information in information theory to measure the dependence between gradient and sensitive information. In information theory, mutual information is the measure of shared information between two random variables, reflecting their degree of dependence. It provides a theoretically provable, universally comparable, and quantifiable measure of the privacy leakage risk of sensitive information from publicly observable data sources.

Based on the adversary threat model analysis and sensitive information in the classification system mentioned above, the corresponding methods of sensitive information in the adversary inference classification system are attribute inference attacks and label inference attacks. In essence, both attack methods use gradient variation rules to distinguish the existence of attributes and labels. The more significant the mutual information between the gradient and the sensitive information, the more accessible for the adversary to infer the sensitive information from the shared gradient (Answer the **Question 4**).

Therefore, a privacy risk score based on normalized mutual information between sensitive information and gradient is introduced to represent the degree of privacy disclosure in the training process of the classification system, as shown in Formula (12):

(12) $${{Risk}_{C,S}^{t,k}}_{\;}=\frac{\sum_{\tau =1}^{m+n}NI\left(S_{\tau };G_{t,k}\right)}{m+n}=\frac{\sum_{h=1}^{h=n}NI\left(F_{h};G_{t,k}\right)+\sum_{j=1}^{j=m}NI\left(y_{j};G_{t,k}\right)\;}{m+n}$$

where ${Risk}_{C,S}^{t,k}$ represents the privacy leakage risk of the FL-based Android malware classification system C for the sensitive information S in the dataset at round t. $NI\left(F_{h};G_{t,k}\right)$, $NI\left(y_{j};G_{t,k}\right)$ represents the normalized mutual information between the functional types of app samples F (*n* types) and the malware tags y (*m* types) of the app and the release gradient $G_{t,k}$ (fitted by app training samples), respectively. By summing the normalized mutual information of all sensitive information S and published gradients $G_{t}$, the average value is taken to describe the overall privacy leakage risk in the classification system C.

If the privacy risk score ${Risk}_{C_{A}}=0$, in the classification system $C_{A}$, it is said that the classification system $C_{A}$ has no privacy leakage risk in terms of sensitive information S (ideal privacy target). If ${Risk}_{C_{A}}>{Risk}_{C_{B}}$, then the privacy leakage risk of the system $C_{A}$ is more serious than that of $C_{B}$ to the sensitive information S it targets.

#### 3.3.2. Calculation Method of Privacy Risk Score

This section gives the calculation method and process of the privacy risk score. First, the mutual information between S and G is defined as Formula (13):

(13) $$\begin{array}{lll} I\left(S;G\right)=H\left(S\right) & - & H\left(S|G\right)\\ & & =-\int_{S}^{\;}p\left(S\right)\log p\left(S\right)dS+\int_{S,G}^{\;}p\left(S,G\right)\log p\left(S|G\right)\mathrm{dSdG}\;\\ & & =\int_{S,G}^{\;}\left(p\left(S,G\right)\log\frac{p\left(S,G\right)}{p\left(S\right)p\left(G\right)}\;\right)dSdG \end{array}$$

The exact computation of $I\left(S;G\right)$ is challenging due to the unknown distribution between sensitive information S and gradient G, and the complex nonlinear relationship between them. Therefore, we need a reliable method for estimating $I\left(S;G\right)$. We adopt the mutual parameterized information estimation approach in the article [22] and represent it as KL divergence, as shown in Formula (14):

(14) $$I\left(S;G\right)=\int_{S,G}^{\;}\left(p\left(S,G\right)\log\frac{p\left(S,G\right)}{p\left(S\right)p\left(G\right)}\;\right)dSdG=D_{KL}\left(P\left(S,G\right)∥P\left(S\right)⨂P\left(G\right)\right)$$

Then, we used the Donsker–Varadhan (DV) of KL divergence to express the expression of mutual information, which became an expected upper bound, and then performed sampling estimation, as shown in Formula (15):

(15) $${\hat{I\left(S_{\;};G\right)}}_{n}=\underset{\theta \subset \Theta }{\sup}E_{{P_{S,G}^{\left(n\right)}}_{\;}}\left[M_{\theta }\right]-\log\left(E_{{P_{S}^{\left(n\right)}⨂{\hat{P}}_{G}^{\left(n\right)}}_{\;}}e^{\left[M_{\theta }\right]}\right)$$

where $M_{\theta }:S\times G\to R$ was based on a neural network (this paper adopted a three-layer MLP) with parameter θ to estimate mutual information. ${M=\left\{M_{\theta }\right\}}_{\theta \subset \Theta }$ is a family of functions that maps two random variables to a constant. θ is optimized from n samples sampled iteratively (in the joint distribution $P_{S,G}^{\;}$ and edge distribution $P_{S}^{\;}$ and ${\hat{P}}_{G}^{\;}$ of S and G) to obtain approximate estimates of mutual information. For details, please refer to the literature [22].

It follows from the definition of mutual information that the range of the value as shown in Formula (16):

(16) $$0\leq I\left(S;G\right)\leq min\left\{H\left(S\right),H(G)\right\}$$

Furthermore, we define the normalized mutual information as shown in Formula (17):

(17) $$NI\left(S;G\right)=\frac{I\left(S;G\right)}{min\left\{H\left(S\right),H(G)\right\}}$$

where $H\left(G\right)=-\int_{G}^{}\left\{f\left(G\right)\log_{2}\left(f\left(G\right)\right)\right\}dG,H\left(S\right)=-\sum_{\tau =1}^{m+n}p\left(S_{\tau }\right)\log_{2}\left(p\left(S_{\tau }\right)\right)$,

${H\left(S\right)\leq log}_{2}(m+n)$ (In the Jensen inequality [42]).

It should be noted that the shared gradient of the Android malware classifier is a high-dimensional variable, which leads to a massive amount of calculation for the estimation of mutual information, and a large number of samples are needed for accurate fitting. Therefore, to reduce the calculated dimension of the privacy risk score in Formula (11), we combine the sensitivity that is more convenient for the calculation to simplify the amount of calculation appropriately.

**Definition 4** (Global sensitivity)**.** *Given function $f:\;D\to R^{d}$, for any two adjacent data sets D and $D^{\prime }$, define the global sensitivity of $S_{f}$ as shown in Formula (18):*

(18) $$S_{f}=\underset{D,D^{\prime }}{max}{∥f(D)-f(D^{\prime })∥}_{2}$$

Among them, $R^{d}$ represents a d-dimensional real vector, $∥f(D)-f(D^{\prime }){∥}_{2}$ is f(D) and $f(D^{\prime })$ between the L2 distance.

Sensitivity is a measure that quantifies how much a slight change in the corresponding input data affects the model’s output. Suppose the gradient is not sensitive to the change in the input data. In that case, it will be more difficult for the adversary to classify the sensitive information of the input data, thus making the inference attack less successful. Therefore, the sensitivity reveals the risk of information leakage from another perspective, and the sensitivity ratio between different layers also approximately reflects the proportion of the privacy risk between them. At the same time, the sensitivity can be constrained by the clipping norm of the gradient, which is easy to calculate [43].

Therefore, we use the ratio of sensitivity of different layers, combined with mutual information, to represent the score of privacy risk, as shown in Formula (19):

(19) $${{Risk}_{C,S}^{t,k}}_{\;}=\frac{1}{m+n}\sum_{\tau =1}^{m+n}\frac{{S_{f}}_{\;}}{{S_{\;}}_{f}^{q}}\cdot NI\left(S_{\tau };{G_{t,k}^{q}}_{\;}\right)$$

It can be seen from Formula (19) that by combining the sensitivity ratio, the calculation of the privacy score only needs to calculate the gradient of the q layer, simplifying the calculation. Since the sensitivity calculation only depends on the classification model trained by the user, the privacy risk it captures is independent of the attack model. It should be emphasized that sensitivity cannot be used as a privacy risk measure because it cannot make an equal comparison between different models and does not meet the principle of applicability. Therefore, we choose such a combination of metrics to avoid the defects of the two types of metrics and comprehensively utilize their advantages to obtain a more reasonable and applicable privacy risk score, as shown in Algorithm 1. The statistical network $M_{\theta }$ in the privacy risk evaluator in our proposed algorithm is a [200×300×150×150×1] MLP network. The computational complexity is $Time∼O\left(200\times 300+300\times 150+150\times 150+150\times 1\right)=O\;\left(127650\right)$. The storage complexity is $Space∼O\left(200\times 300+300+300\times 150+150+150\times 150+150+150\times 1+1\right)=O\left(128251\right)$.
**Algorithm 1: Privacy Risk Evaluation Mechanism of FedDroidMeter****input**Gradient of client k in round t is $G_{t,k}^{\;}$, the corresponding batch of client’s app samples B and sensitive information S=(F,y), the q-layer of gradient selected for calculating risk.**Output**Privacy risk score ${Risk}_{C,S}^{t,k}$ of the gradient $G_{t,k}$ on client k.1:Calculate mutual information of sensitive information $I\left(S;G_{t,k}^{}\right)$ on the client k by Formular (15).2:Calculate normalized mutual information of sensitive information $NI\left(S;G_{t,k}^{}\right)$ on the client k by Formular (17).3:Calculate sensitivity of the entire gradient $S_{f}$ and the sensitivity of the q-layer of gradient $S_{f}^{q}$ by Formular (18).4:Calculate privacy risk score ${Risk}_{C,S}^{t,k}$ by Formular (19).5:Return privacy risk score ${Risk}_{C,S}^{t,k}$.6:**end**

## 4. Results

In this section, we first introduce the datasets and data settings used in the experiment. Then, we give the relevant setup of the experiment, including the model structure and training details. Next, we introduce metrics to evaluate the effectiveness and applicability of privacy scores. Then, we systematically evaluate the effectiveness of privacy scoring on the Androzoo dataset and baseline classification models; Then, we evaluate the applicability of privacy scores in different use cases by setting different hyperparameters of the model and privacy parameters. Finally, we discuss the experimental results and preliminarily analyze how the privacy risk changes under various hyperparameters of the model and privacy parameter settings.

### 4.1. Dataset

To test the performance of the model and algorithm, we built a dataset from the open-source project Androzoo [44], which has about 60,000 apps (9 categories) for the Android malware classification task. At the same time, we extract app functional categories (10 categories) from the metadata of APK files for attribute labeling, as shown in Table 4. We use 10-fold cross-validation, randomly generating the test set from 1/10 of the users. The reported results correspond to an average of 20 replicates per experiment (Inferred attack and evaluation of risk score). The privacy inference model uses training data from the whole app datasets as background knowledge.

### 4.2. Model and Training Setup

#### 4.2.1. The Setup of FL-Based Android Malware Classification System

To verify the effectiveness and applicability of the proposed privacy score, we choose FedIIoT [14] and FedRAPID [16], two baseline FL-based Android malware classification systems (without defense), as the objects to evaluate the privacy risk. FedIIoT uses a six-layer FC and SoftMax layer classifier structure and uses API, permission, and intention as input features. FedRAPID uses a classifier structure of five Conv layers and four FC layers to take the grayscale image of the binary transformation of the application as the input features.

Among them, the main task of the classifier is set as Android malware classification (nine categories, which are also used as the target of label inference), and the sensitive attributes are set as 10 app function categories.

For the FL training, we set ten clients, overall training round T = 320, local training epoch = 1 for clients, and the optimizer of SGD. We evaluated the experiment on an Intel Golden 6240 CPU and an NVIDIA A100 GPU. FL-based Android malware classifiers and privacy risk evaluation methods are implemented in PyTorch.

#### 4.2.2. Attack Model

Aiming at the privacy risk of users’ sensitive information in the FL-based malware classification framework, we choose the attribute inference attack model proposed in [5,23,30] to infer the function types of applications in the training samples using shared gradients. The label inference attack model proposed in [5,22,45] (we take the label as the attribute of the target as input) is selected to infer the type of malware labels in the training samples using the shared gradient.

The auxiliary dataset of the adversary is whole app datasets, and the attack model is trained (10-fold cross-validation) using the updated gradients generated (generated on the Android malware classifier) from the auxiliary dataset. Finally, the attack model tests the effect of its inference attack on the gradients generated from the attacked client’s data.

#### 4.2.3. Evaluation Methods and Metrics

In this section, we introduce the metrics to evaluate the effect of privacy inference attacks, the effect of malware classification, and the effectiveness (principles (1) and (2)) and applicability (principle (3)) of privacy scores.

The first metric we evaluate is the effect of the attacks, which is the threat degree by privacy inference attack, which is the average F1-score (20 inference experiments) of sensitive information inferred by inference attack. The description of the F1-score is shown in Table 5. Among them, we define each type of sensitive information (nine types of malware categories and ten types of app function categories) as positive samples and the other categories as negative samples. Then, the average value of each category evaluation index is taken as the final evaluation index. Similarly, the evaluation metric for malware type classification (nine malware classes) is also the F1-score. We define each classified malware class as a positive sample and the other classes as negative samples.

In a real scenario, the configuration of the attacker is not known. Therefore, to verify the worst-case privacy risk, we choose the maximum F1-score of the inferred attack as the measure of the attack effect in the test results of various tuned attack models (The maximum F1-score of each type of label attack and attribute attack on all clients is averaged).

Previous papers [27,35] have proposed using the inference attack of attack classifier F1-score to measure the privacy risk of input because it corresponds to the probability of sensitive information being successfully inferred in the gradient. Therefore, we take the average F1-score of the attack models as the ground truth of privacy risk. Suppose the privacy risk score corresponds to the risk of leaking sensitive information about the sample in the gradient. In that case, the value of the privacy risk score (and the trend of change) in each batch and the F1-score of the corresponding attack model are closely related. Therefore, we evaluated the effectiveness of different privacy evaluation mechanisms (in the unified model) by calculating the Pearson coefficient between the privacy risk score in the training process and the inferred F1-score.

Finally, we evaluate its applicability using the Pearson coefficient between the privacy score and the inference F1-score of the inferred attack in different model parameters, different model structures/inputs, and different settings of privacy parameters (to generate different use cases).

### 4.3. Test Effectiveness of the Privacy Risk Score

#### 4.3.1. Test the Effectiveness of Privacy Risk Scores in Time Dimensions

In this section, to verify the effectiveness of the privacy score of FedDroidMeter in this paper, we systematically evaluate the privacy risk of the baseline federal malware classifier, FedIIoT [14], and FedRAPID [16], using multiple types of baseline attack models. The proposed method’s effectiveness is compared with the state-of-the-art work SHAPR [34] and the clustering distance-based method [40] in the same simulation configuration.

SHAPR measures privacy risk based on the model utility of training samples. Clustering distance-based methods apply the clustering distance of training samples for privacy risk evaluation.

**Figure 5:**
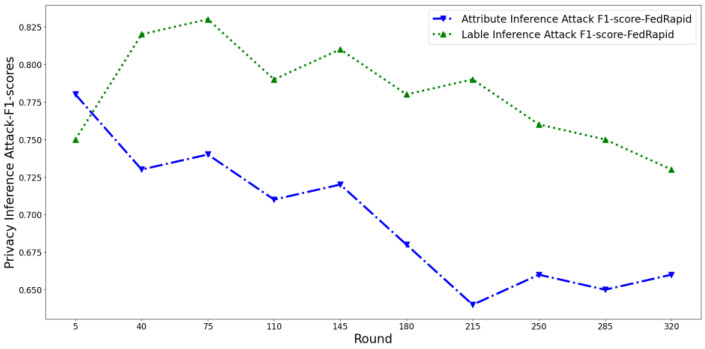
Privacy inference attack F1-scores in different rounds of training (FedRAPID).

**Figure 6:**
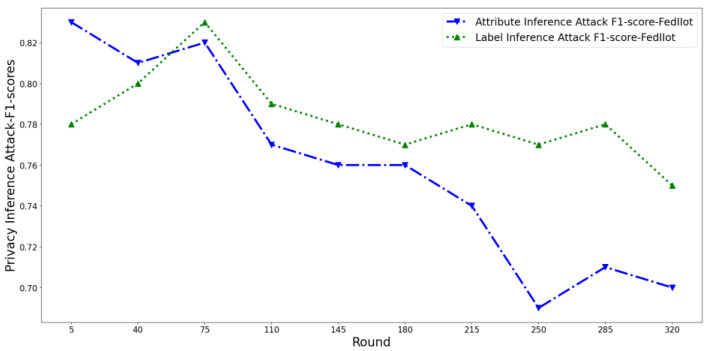
Privacy inference attack F1-scores in different rounds of training (FedIIot).

At the same time, we perform several attack models described in Section 4.2.2 that pose privacy threats to users to conduct inference attacks and count the average F1-score value of the attack classifier. Then, we plot the privacy risk scores output by the three scoring mechanisms and the corresponding inference attack F1-score, as shown in Figure 5 and Figure 6. For ease of display, the privacy score is properly normalized.

It can be seen that in the early training phase, the F1-score of the attribute inference attack shows a slow decreasing trend, the F1-score of the label inference attack shows an increasing trend, and the overall F1-score shows an increasing trend.

In the middle of training, the F1-score of the attribute inference attack shows a slow decreasing trend, while the F1-score of the label inference attack gradually reaches the peak and starts to decrease. The overall F1-score showed a gradual decline trend.

In the later stage of training, the F1-score of both the attribute and label inference attacks gradually flattens out. The overall F1-score also gradually leveled off.

This tendency is caused by the change in model state during training. At the beginning of training, the classification model is more sensitive to app samples because it has “seen” fewer samples and “remembers” more information in app samples. With the increase of training samples, the model’s generalization ability is enhanced, so the memory ability of the sample information gradually decreases and tends to be stable. Therefore, the opponent’s difficulty in inferring attribute information from the gradient is gradually increased, i.e., the privacy risk of attribute information is gradually reduced. In addition, the fitting degree of the model to the label category was low at the beginning of the training and then gradually increased and tended to be flat. Therefore, the opponent’s difficulty inferring the label from the gradient also experienced a change law of first increasing and then decreasing.

**Figure 7:**
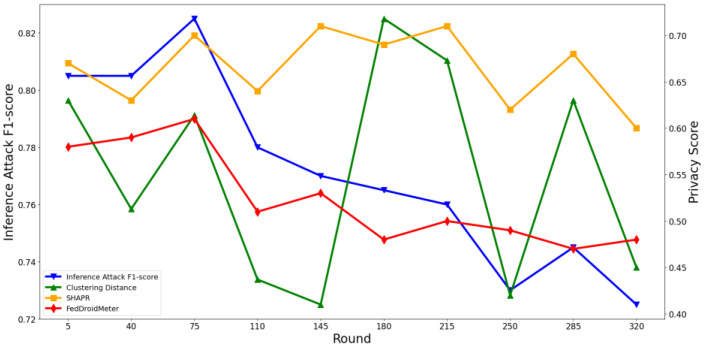
Privacy risk scores in different rounds of training (FedIIot).

**Figure 8:**
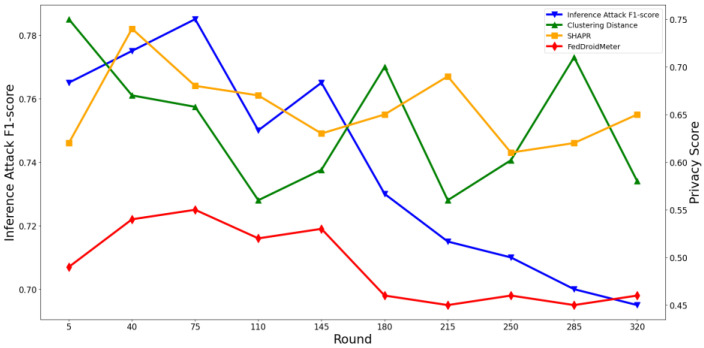
Privacy risk scores in different rounds of training (FedRAPID).

At the same time, as can be seen in Figure 7 and Figure 8, the privacy risk score of our proposed FedDroidMeter is closely related to the F1-score of inference attack, which accurately tracks the changing trend of privacy leakage risk (Pearson correlation coefficient is as high as 0.92). As for SHAPR, it tracks the changing trend of privacy leakage risk to some extent, but the correlation is lower than that of FedDroidMeter, and the Pearson coefficient is only 0.43. This is because SHAPR focuses on the memory degree of the training sample itself (x, y) in the classification model but does not pay attention to the memory degree of the sensitive information (malware label y and function type F) in the fine-grained. Therefore, it does not capture the essence of the privacy risk of the system, and the score’s effectiveness is poor. The clustering distance-based method has the lowest performance (Pearson coefficient is only 0.24). This is because it only focuses on the distribution characteristics of the sample set and does not consider the change in the model’s state in the training process, so the evaluation effect is poor.

#### 4.3.2. Test the Effectiveness of Privacy Risk Score in the Spatial Dimension

**Figure 9:**
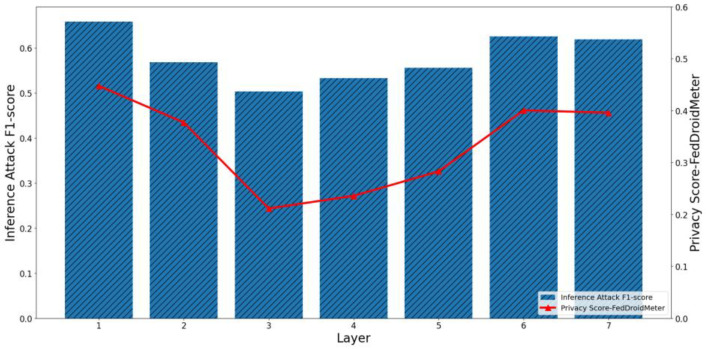
Privacy risk scores in different layers of gradient (FedIIot).

**Figure 10:**
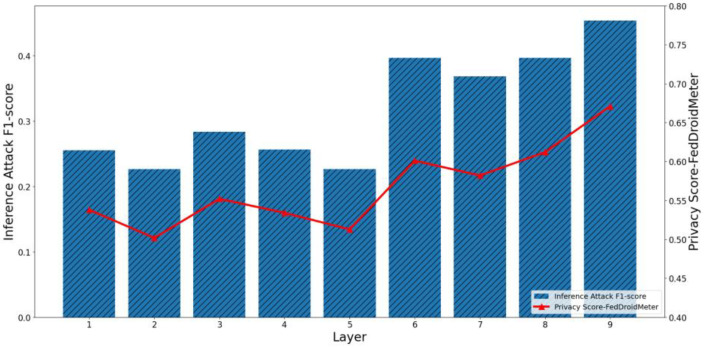
Privacy risk scores in different layers of gradient (FedRAPID).

Furthermore, we evaluate the effectiveness of our scoring method in estimating the privacy risk between different layers of the neural network. It should be pointed out that SHAPR and the clustering distance-based method only support the overall privacy risk evaluation of the model rather than the fine-grained privacy risk evaluation of different layers. Therefore, we only show the effects of FedDroidMeter’s privacy risk score. As shown in Figure 9 and Figure 10, we present the privacy risks of different layers of the two classification models’ gradients and the corresponding privacy inference attack F1-score.

In the FedIIoT, the F1-score of privacy inference attacks near the first and last FC layers is high, while the F1-score of other layers is low. In the FedRAPID, the F1-score of privacy inference attacks near the last Conv layer, and the output FC layer is higher. Meanwhile, in the gradient of different layers of the two models, the privacy risk score of FedDroidMeter proposed by us closely tracked the F1-score (Pearson correlation coefficient is as high as 0.908) of the privacy inference attacks.

In addition, we analyzed the different processing mechanisms of the FC layer and Conv layer to the characteristics of malware that lead to the difference in the risk distribution. In the FedRAPID, the first several Conv layers convolved the features of malware (local and separate), so a complete feature view could not be obtained. Ultimately, all local features are integrated by the FC layer after the Pooling layer so that a more complete feature view is obtained, and higher privacy risks are encountered. However, in the FedIIoT, the first FC layer encodes all input features uniformly (fully connected), thus offering a more complete view of the original sensitive information. Hence, this layer has a higher privacy risk. In addition, the last layer of the two classification models is the FC layer. As the output layer, the FC layer has more label information, so the privacy risk is high.

Therefore, our proposed FedDroidMeter is still effective for privacy risk evaluation at different gradient layers (i.e., spatial dimension).

To sum up, the proposed privacy risk scoring mechanism, FedDroidMeter, can effectively capture privacy risks in the training process and meet principles (1) and (2).

### 4.4. Test the Applicability of Privacy Risk Scores

In this section, we apply the privacy risk scoring mechanisms to different use cases for an equal privacy risk comparison to verify their applicability.

#### 4.4.1. Test the Applicability of Privacy Risk Scores to Different FL Parameters

**Figure 11:**
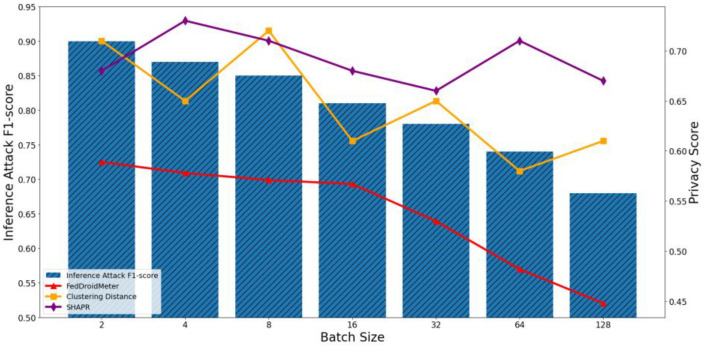
Privacy risk scores in different settings of batch size (FedRAPID).

**Figure 12:**
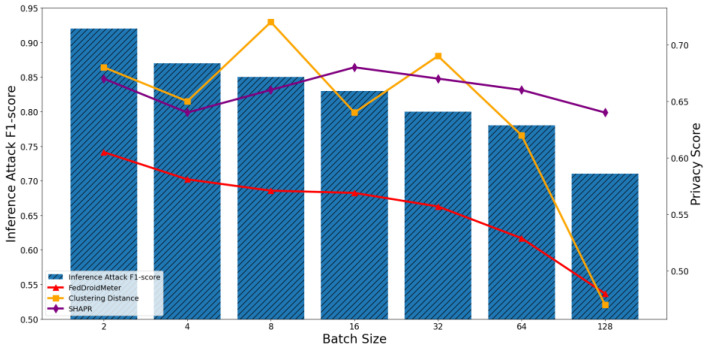
Privacy risk scores in different settings of batch size (FedIIot).

**Figure 13:**
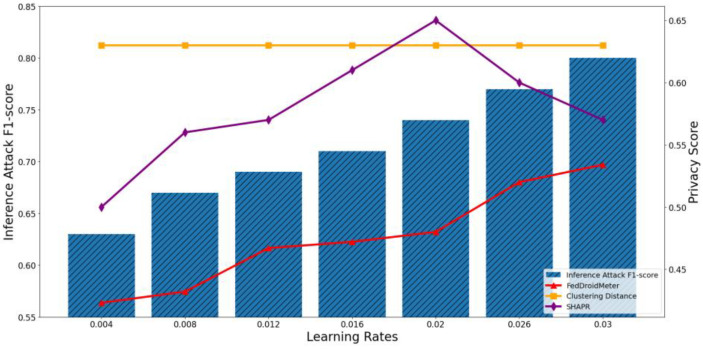
Privacy risk scores in different settings of learning rate (FedRAPID).

**Figure 14:**
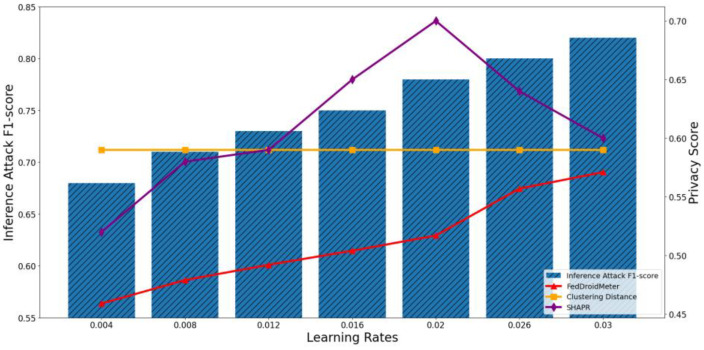
Privacy risk scores in different settings of learning rate (FedIIot).

**Figure 15:**
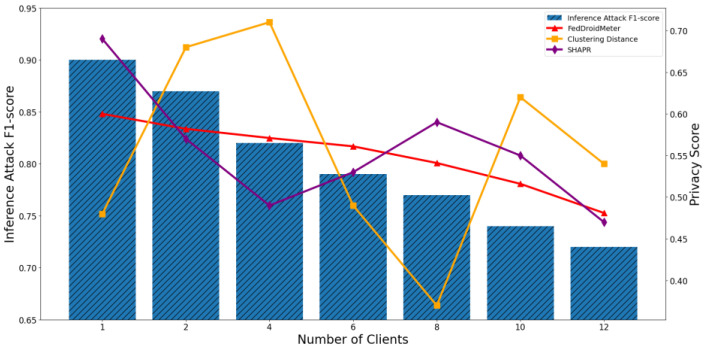
Privacy risk scores in different settings of number of clients (FedRAPID).

**Figure 16:**
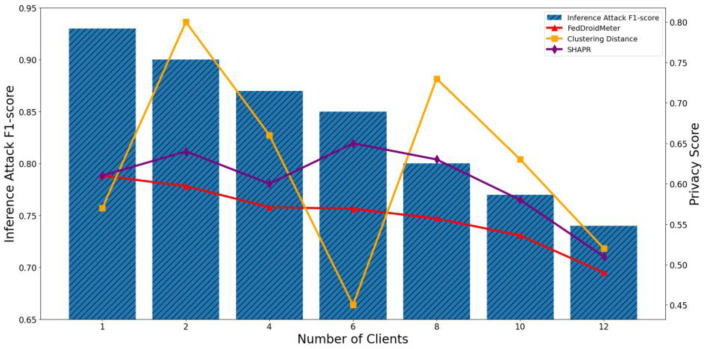
Privacy risk scores in different settings of number of clients (FedIIot).

In our experiments, we construct three types of different use cases with different FL hyperparameter settings, which are different batch sizes [2, 4, 8, 16, 32, 64, 128], different learning rates [0.004, 0.008, 0.012, 0.016, 0.02, 0.026, 0.03], and the different number of clients [1, 2, 4, 6, 8, 10, 12]. To compare the applicability of privacy scoring mechanisms to different use cases, we perform privacy risk scoring and privacy inference attacks on these use cases and analyze their correlation. The experimental results are shown in Figure 11, Figure 12, Figure 13, Figure 14, Figure 15 and Figure 16, where each column represents a different evaluation use case and its corresponding F1-score of privacy inference attacks, and each line represents the risk score of the corresponding use case with different privacy risk scoring mechanisms.

It can be seen that when the batch size increases from 2 to 128, the F1-score of privacy inference attacks decreases continuously, as shown in Figure 11 and Figure 12. This is because the gradient generated by more samples (a local average) dilutes the content of each category of sensitive information in a single sample in the gradient, resulting in a smaller probability of its leakage. In addition, more training samples make the generalization performance of the model of each round of training better and reduce the degree of “memory” of sensitive information.

The Pearson coefficient of privacy score based on clustering distance and privacy inference attack F1-score was 0.73, lower than that of FedDroidMeter (Pearson coefficient was 0.92). This is because the privacy score based on clustering distances captures changes in sample distances caused by the increase in the batch and, in turn, changes in privacy risks along the way. However, because it does not consider the content of sensitive information in samples, the effectiveness of the evaluation is inferior to that of FedDroidMeter. In addition, since the score of SHAPR is based on the average utility of each sample, it is less affected by the batch size. Therefore, SHAPR cannot effectively capture the difference in privacy risks in different batch-size use cases.

The F1-score of the privacy inference attack keeps increasing when the learning rate increases from 0.004 to 0.03, as shown in Figure 13 and Figure 14. This is because a more significant learning rate makes the gradient learn more about the samples in each training batch, potentially containing more sensitive information.

The Pearson coefficient of the F1-score of privacy inference attacks and SHAPR was 0.62, lower than that of FedDroidMeter (Pearson coefficient was 0.95). This is because the SHAPR score is based on the utility of the sample to the model. In increasing the learning rate, the model utility gradually increases within a reasonable range. Then, the training accuracy decreases due to the high learning rate, resulting in a decrease in the SHAPR score. However, in the process of increasing the learning rate, because the learning degree of the model to the sample increases, the risk of leakage of sensitive information from the gradient increases. FedDroidMeter’s risk score is based on the mutual information of sensitive information in the gradient, thus correctly tracking this risk trend and giving a more effective risk score.

In addition, the privacy score based on clustering distance only considers the distance of samples. It is not affected by the learning rate, so it is constant and cannot effectively assess the privacy risk of these use cases.

It can be seen that when the number of clients increases from 1 to 12, the F1-score of privacy inference attacks decreases continuously, as shown in Figure 15 and Figure 16. This is because the aggregated classification model on more clients has a lower degree of fit for each single client training sample, making the locally uploaded gradient incorporate less sensitive information about the local training set. The Pearson coefficient of the F1-score of privacy inference attacks and SHAPR was 0.86, lower than that of FedDroidMeter (Pearson coefficient was 0.93). This is because SHAPR scores are based on the utility of the sample to the model. In the process of increasing the number of trained clients, the model’s generalization ability is gradually enhanced. Within a specific range, the utility of the sample to the model is increased. However, with the continuous increase of clients, the fitting degree of the model to the sample set of a single client becomes lower and lower, which exceeds the effect of the increase of model utility brought by the increase of generalization performance. Therefore, the SHAPR score begins to decline. However, with the increase in the number of clients, the sensitive information about the clients in the model’s gradient is constantly “diluted,” so the difficulty of obtaining sensitive information from the opponents is constantly increased. On the other hand, FedDroidMeter’s risk score is based on the mutual information of sensitive information in the gradient and thus correctly tracks this risk trend. In addition, the privacy score based on clustering distance does not consider the changes in the model state during the process of client increase, so the privacy risk of these use cases cannot be effectively assessed, and the Pearson coefficient is only 0.21.

#### 4.4.2. Test the Applicability of Privacy Risk Score to Different Privacy Parameters

In addition, we build two different types of use cases for different privacy protection parameter settings: different noise scale = [0.1, 0.5, 1, 1.7, 2.2, 2.8, 3.5], and different clipping norm of gradient [1, 3, 6, 8, 10, 15, 20]. They are the critical parameters in the differential privacy protection mechanism, which adds noise to the model parameters to limit the adversary’s inference to sensitive information.

**Figure 17:**
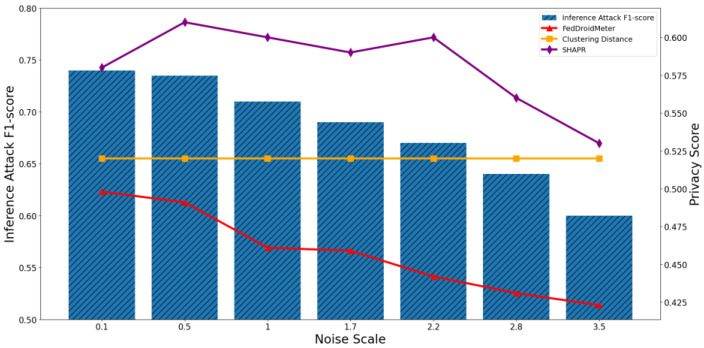
Privacy risk scores in different settings of noise scale (FedRAPID).

**Figure 18:**
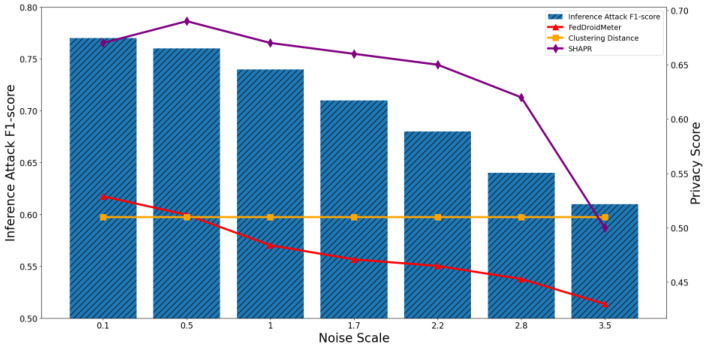
Privacy risk scores in different settings of noise scale (FedIIot).

As the noise scale increases from 0.1 to 3.5, the F1-score of the privacy inference attack decreases continuously, as shown in Figure 17 and Figure 18. Because the increase of added noise disturbs the distribution of sensitive information in the gradient, it prevents the opponent from inferring sensitive information.

The Pearson coefficient of SHAPR’s privacy score and privacy inference attack F1-score was 0.83, lower than that of FedDroidMeter (Pearson coefficient 0.93). This is because the SHAPR score is based on the utility of the sample to the model. When the noise increases within a specific range, it increases the accuracy of the model (such as increasing the generalization of the model and helping to escape from the local saddle point in training quickly), so the model utility gradually increases, and the SHAPR score increases. Then, since the noise variance gradually exceeds the original gradient’s variance, the model utility decreases, and the SHAPR score decreases. However, in the process of increasing noise, the privacy risk is constantly reduced due to the adversary’s increasing difficulty in obtaining sensitive information in the gradient. FedDroidMeter’s risk score is based on the mutual information of sensitive information in the gradient, thus correctly tracking this risk trend and giving a more effective risk score.

In addition, privacy scores based on clustering distances only consider the distance of samples. They are not affected by the noise applied on the gradient, so they are constant and cannot effectively assess the privacy risk of these use cases.

**Figure 19:**
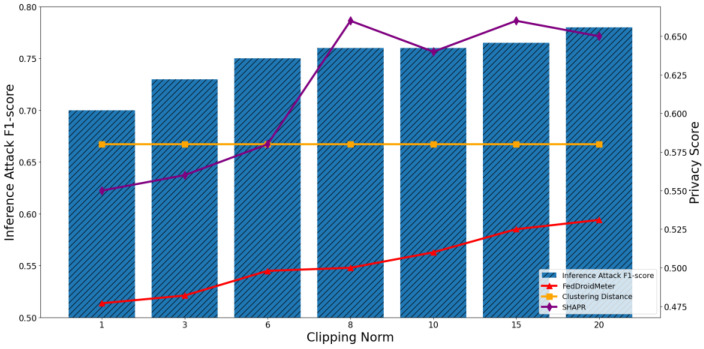
Privacy risk scores in different settings of the clipping norm (FedRAPID).

**Figure 20:**
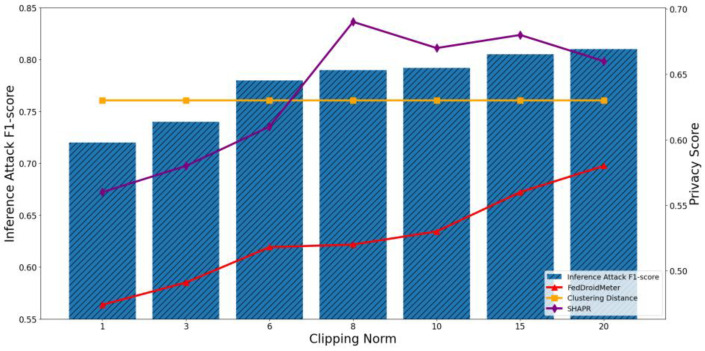
Privacy risk scores in different settings of the clipping norm (FedIIot).

In Figure 19 and Figure 20, we can observe that when the clipping norm of the gradient increases from 1 to 20, the F1-score of the privacy inference attack first gradually increases and then levels off. This is because as the norm of the gradient clipping increases, the sensitivity of the gradient increases, so the adversary’s ability to infer sensitive information becomes more accessible. Furthermore, when the trimmed gradient gradually approaches the original gradient, the changing trend of privacy leakage risk becomes stable.

The Pearson coefficient of the F1-score of privacy inference attacks and SHAPR was 0.86, lower than that of FedDroidMeter (Pearson coefficient was 0.93). Especially in the range of 6–20, the SHAPR score has a poor ability to track privacy risks. This is because the small norm within the appropriate range plays a certain role in enhancing the model’s generalization and improving the model’s practicability, resulting in the score of SHAPR does not monotonically increase with the increase of the gradient clipping norm. However, with the increase of gradient clipping norm, the adversary’s ability to acquire sensitive elements is improved. On the other hand, FedDroidMeter’s risk score is based on the mutual information of sensitive information in the gradient and thus correctly tracks this trend. In addition, since the change in gradient clipping norm does not affect the clustering distance of training samples, the privacy score based on clustering distance is constant. It cannot capture the difference in privacy risk between different use cases.

#### 4.4.3. Test the Applicability of Privacy Risk Scores to Different Models

**Figure 21:**
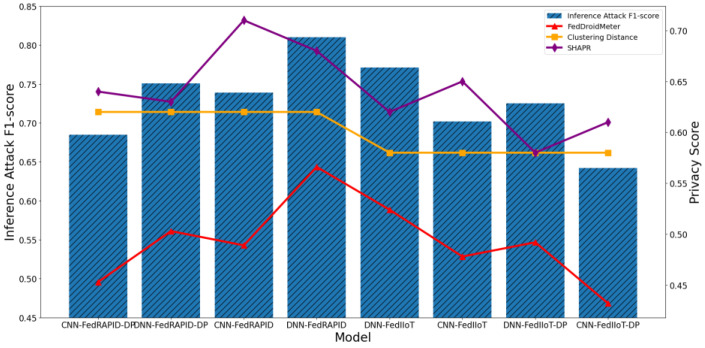
Privacy risk scores in different classification models.

Furthermore, to test the suitability of the privacy scoring mechanism for use cases with different model architectures and input characteristics, we exchange the input characteristics of (FedIIot) and FedRAPID and add Gaussian noise (noise scale = 1.5) as a supplement to the four sets of use cases. The results of the test are shown in Figure 21.

By comparing the F1 scores of privacy inference attacks of CNN and the DNN (FC) model, it can be found that DNN has a higher risk of privacy disclosure. According to our preliminary analysis, this is because the feature processing of CNN in the first few layers is local and separated, resulting in the dispersion of sensitive information in the gradient, which is difficult for opponents to use for inference.

By comparing the F1 score of the privacy inference attack of bytecode and the input feature of API-Intent-Permission, we can find that the input feature of API-Intent-Permission has a lower risk of privacy disclosure. We hypothesize that this is because the input feature of API-Intent-Permission contains less semantic information about the app’s functional category (sensitive information) than the bytecode feature and thus is more easily recognized by adversaries.

The F1 score of privacy inference attacks of the model shows that the F1 score of privacy inference attacks of DNN is higher.

We then analyzed the correlation between the privacy risk scores for six use cases and the F1 scores for privacy inference attacks, as shown in Figure 21. The Pearson coefficient of SHAPR’s privacy score and privacy inference attack F1-score was 0.38, lower than that of FedDroidMeter (Pearson coefficient 0.96). This is because the SHAPR score is based on the utility of the sample to the model, and the utility values between different model structures CNN and DNN cannot be compared equally. In addition, the utility values of the model between use cases with different input characteristics cannot be compared equally, which leads to the poor applicability of SHAPR’s privacy risk score to different use cases. Finally, the privacy risk scoring mechanism based on clustering distance cannot capture the differences in the model structure, so it is unsuitable for different use cases.

A synthesis of the above experimental analysis, the proposed privacy risk evaluation mechanism, and FedDroidMeter can apply well to different evaluation use cases and meet the three principles.

## 5. Discussion

In the above experiments, we tested different FL parameters, different privacy parameters, different model settings, and input features to prove the effectiveness and applicability of our proposed privacy risk evaluator FedDroidMeter. By comparing the evaluation effect of the two baseline evaluation mechanisms, the evaluation method proposed in this paper can better capture the essential cause of privacy disclosure in the system. Evaluation methods based on coarse-grained model utility (without taking sensitive information into account) or without considering the model state’s impact are insufficient to provide effective evaluation results.

In addition, through the evaluation test of multiple settings, we preliminarily summarized the rules of privacy risk in an FL-based Android malware classification system: (1) When the model performs normal classification function, privacy risk is negatively correlated with learning rate and gradient clipping norm; the number of clients, batch size, and noise scale are negatively correlated. (2) Regarding model structure and input characteristics, CNN seems to have less leakage risk than DNN; Bytecode seems to have less privacy risk than API-Intent-Permission. The more rigorous experimental and theoretical proof is beyond the scope of this paper, which will be considered in future research work. (3) The distribution of privacy risks in space and time is uneven. In spatial dimensions, the first FC layer near the last classification layer and the front end have the highest privacy risk. In the time dimension, privacy risk presents a trend of first increasing and then decreasing, which is related to the fitting state of the model.

It should be noted that our privacy risk score is calculated from the normalized information of the sensitive information and the gradient. It is essentially the amount of information, not the standard probability value. Therefore, our privacy score is not directly equal to the F1 score of the opponent’s inferred attack but is positively correlated with each other. How to strictly track the F1-score of adversary’s inferred attacks, i.e., how to express privacy score as a strict probability representation (probability upper bound) of the F1-score of adversary’s inferred attacks, is beyond the scope of this paper and will be the direction of improvement in the future.

Finally, in light of these experimental findings, we highlight the significant privacy risks in existing FL-based Android malware classification systems. At the same time, it also emphasizes the necessity of privacy assessment and protection measures based on scene characteristics and user privacy needs.

## 6. Conclusions

In this work, we propose FedDroidMeter, a privacy risk evaluation framework based on normalized mutual information according to actual scenario characteristics and user privacy requirements, to evaluate the risk of the indirect information leakage of FL-based Android classifiers. Experimental results on the Androzoo dataset and the baseline FL-based malware classification model show that our proposed FedDroidMeter can effectively measure the privacy risk of classification models. We also warn about the severity of privacy risks in existing federal malware classification frameworks (privacy inference F1-scores up to 0.8). Furthermore, we evaluate the privacy risk of different use cases with different FL parameters and privacy protection parameters and verify the applicability of our risk evaluation method to different use cases. At the same time, we observe and analyze the law of privacy risk of existing classifiers in the setting of different parameters in the discussion of experimental results. The experimental results and findings highlight the importance of providing a systematic privacy risk evaluation framework for FL-based Android malware classifiers and provide empirical and theoretical grounds for developers to investigate targeted defense methods.

The privacy scoring mechanism proposed in this paper is aimed at the indirect privacy threat of privacy inference attacks based on neural networks. The evaluation of other unknown types of privacy threats is out of the scope of this paper and will be used as the expansion direction of FedDroidMeter in the future. At the same time, we plan to explore ways to optimize the computational efficiency of our privacy scoring mechanism to facilitate its deployment and use in federal malware classification systems. Furthermore, we explore the privacy defense mechanism based on privacy score.

## Figures and Tables

**Table 1:** Summarization of previous privacy evaluation methods for federated learning.

Ref	Methods	Limitation	Attack-Agnostic	User- Oriented	Equal Comparison
[30]	The results of performing member inference attacks.	These methods depend on the results of specific attack settings and cannot provide effective results evaluation for users.	-	-	-
[31]	Mean square error (MSE) between the reconstructed and original images.		-	-	-
[33]	The results of multiple types of privacy inference attacks.		-	-	-
[32]	The results of multiple types of privacy inference attacks.		-	-	-
[34]	Based on the Shapley value of samples.	These methods are not targeted to evaluate the sensitive information related to user privacy and cannot capture the essential cause of privacy disclosure.	-	-	-
[37]	Maximum information leakage.		√	-	-
[38]	Total variation distance.		√	-	-
[35]	Probability of a single sample in the target model training set.		√	-	√
[36]	Fisher Information.		√	-	-
[39]	Case-by-case privacy accounting for DP.		√	-	-
[40]	K-means clustering distance.		√	-	-
Proposed FedDroidMeter	Normalized mutual information of the user’s sensitive information.	-	√	√	√

**Table 2:** Description of principles for design goal.

Principle	Description
User-oriented privacy requirements	The resulting privacy risk score must relate to the greatest likelihood that the sensitive information (the user cares about) will be inferred to succeed.
Attack-agnostic	The metrics should capture the root cause of any perceived attack success. The privacy risk score it calculates must be independent of the particular attack model, allowing the score to assess the privacy risk from different or future inferred attacks.
Equal comparison between different use cases	The privacy risk score obtained from the evaluation should be applied to different use cases for equal comparison.

**Table 3:** List of abbreviations and mathematical symbols used.

Abbreviation and Symbol	Explanation
FL	Federated learning
DL	Deep learning
ML	Machine learning
$D_{k}$	Local dataset of app sample
$D_{k}$	Size of local dataset
W	Global classification model
F(W)	Global loss function
$F_{k}(W)$	Local loss function
X	Feature space of Android malware
Y	Category space of Android malware
x	Feature of Android malware
y	Category of Android malware
F	The functional class of the training app samples
$G_{\;}$	Gradient of initialization parameters
S	Sensitive information in the system
I	Mutual information
NI	Normalized mutual information
Risk	Privacy risk score
$S_{f}$	Global sensitivity
q	$\mathrm{Layer}\;\mathrm{of}\;\mathrm{the}\;\mathrm{gradient}\;G_{}$

**Table 4:** Description of the experimental dataset.

Category of Malware	Quantity	Category of App Function	Quantity
Benign	29,977	Photography	5104
Malicious	23,760	Books	6381
Adware	4728	Sports	4632
Trojan	3638	Weather	5396
Riskware	3193	Game	7142
Ransom	4322	Finance	5665
Exploit	1825	Health And Fitness	4745
Spyware	2476	Music and Audio	6043
Downloader	4023	Shopping	3874
Fraudware	3776	Communication	4824

**Table 5:** Performance Metrics.

Performance Metrics	Calculation Formula
Precision	$PRE=\frac{TP}{TP+FP}$
Recall	$REC=\frac{TP}{TP+FN}$
F1-score	$F1=\frac{2\times PRE\times REC}{PRE+REC}$

## Data Availability

Not applicable.
